# Detection of drug resistant *Mycobacterium tuberculosis* by high-throughput sequencing of DNA isolated from acid fast bacilli smears

**DOI:** 10.1371/journal.pone.0232343

**Published:** 2020-05-08

**Authors:** Mazhgan Rowneki, Naomi Aronson, Peicheng Du, Paige Sachs, Robert Blakemore, Soumitesh Chakravorty, Shawn Levy, Angela L. Jones, Geetika Trivedi, Sheilla Chebore, Dennis Addo, Denis K. Byarugaba, Panganani Dalisani Njobvu, Frederick Wabwire-Mangen, Bernard Erima, Eric S. Ramos, Carlton A Evans, Braden Hale, James D. Mancuso, David Alland

**Affiliations:** 1 Department of Medicine, Rutgers New Jersey Medical School, Newark, New Jersey, United States of America; 2 Department of Medicine, Uniformed Services University of the Health Sciences, Bethesda, Maryland, United States of America; 3 Office of Advanced Research Computing, Rutgers University, Newark, New Jersey, United States of America; 4 Genomics Services Laboratory, HudsonAlpha Institute for Biotechnology, Huntsville, Alabama, United States of America; 5 Kenya Medical Research Institute, U.S. Army Medical Research Directorate-Africa, Kericho, Kenya; 6 Ghana Armed Forces Tuberculosis Control Program, 37 Military Hospital, Accra, Ghana; 7 Makerere University Walter Reed Project, Kampala, Uganda; 8 Zambia Defence Force Medical Services, Maina Soko Military Hospital, Lusaka, Zambia; 9 Innovation For Health And Development, Laboratory for Research and Development (IFHAD), Universidad Peruana Cayetano Heredia, Lima, Peru; 10 Innovacion Por la Salud Y el Desarollo (IPSYD), Asociación Benéfica Prisma, Lima, Peru; 11 Infectious Diseases & Immunity, Wellcome Trust Imperial College Centre for Global Health Research, London, United Kingdom; 12 Naval Health Research Center, Defense Health Agency, San Diego, California, United States of America; 13 University of California San Diego, La Jolla, California, United States of America; 14 Armed Forces Health Surveillance Branch, Silver Spring, Maryland, United States of America; Institut de Pharmacologie et de Biologie Structurale, FRANCE

## Abstract

**Background:**

Drug susceptibility testing for *Mycobacterium tuberculosis* (MTB) is difficult to perform in resource-limited settings where Acid Fast Bacilli (AFB) smears are commonly used for disease diagnosis and monitoring. We developed a simple method for extraction of MTB DNA from AFB smears for sequencing-based detection of mutations associated with resistance to all first and several second-line anti-tuberculosis drugs.

**Methods:**

We isolated *MTB* DNA by boiling smear content in a Chelex solution, followed by column purification. We sequenced PCR-amplified segments of the *rpoB*, *katG*, *embB*, *gyrA*, *gyrB*, *rpsL*, and *rrs* genes, the *inhA*, *eis*, and *pncA* promoters and the entire *pncA* gene.

**Results:**

We tested our assay on 1,208 clinically obtained AFB smears from Ghana (n = 379), Kenya (n = 517), Uganda (n = 262), and Zambia (n = 50). Coverage depth varied by target and slide smear grade, ranging from 300X to 12000X on average. Coverage of ≥20X was obtained for all targets in 870 (72%) slides overall. Mono-resistance (5.9%), multi-drug resistance (1.8%), and poly-resistance (2.4%) mutation profiles were detected in 10% of slides overall, and in over 32% of retreatment and follow-up cases.

**Conclusion:**

This rapid AFB smear DNA-based method for determining drug resistance may be useful for the diagnosis and surveillance of drug-resistant tuberculosis.

## Background

Globally, tuberculosis (TB) remains one of the top ten causes of death [1]. TB treatment has been complicated by a rise in drug resistance, with treatment success rates of approximately 55% for rifampicin-resistant (RR) and multidrug-resistant (MDR) TB (defined as resistance to both isoniazid (INH) and rifampicin (RIF)), and approximately 34% for extensively drug-resistant (XDR) TB (defined as MDR plus resistance to the fluoroquinolone (FQ) antibiotics and second-line injectable drugs) [1]. Commercially-available molecular assays, such as the GeneXpert^®^ MTB/RIF (Cepheid, Sunnyvale, CA) and the GenoType MDRTBplus (Bruker-Hain Diagnostics, Nehren, Germany), have shown excellent sensitivity and specificity for detecting RIF and RIF and INH resistance respectively. However, cost and technical complexity of these tests may be prohibitive in resource-limited areas. Additionally, these assays do not identify pyrazinamide (PZA) resistance, which is strongly associated with the success or failure of World Health Organization (WHO)-recommended shortening regimens [2].

Widespread use of TB drug susceptibility testing (DST) would aid in prevention and treatment of drug-resistant TB. Universal DST for at least RIF is recommended in all TB cases [3]. DST enables expanded use of standardized short course MDR treatments, which require susceptibility testing to major components of treatment before treatment initiation [2]. Broad use of culture or molecular-based DST methods has been difficult to achieve in resource-limited settings. Global surveillance data indicates that DST for RIF was only performed for 24% of new TB cases and 70% of previously treated cases in 2017 [1]. DST for PZA has been especially difficult to perform because phenotypic testing is technically challenging and nucleic acid amplification based tests (NAAT) must be able to identify hundreds of different mutations to effectively detect most cases of PZA resistance [4]. Performing DST at centralized locations might improve access to some types of drug susceptibility results, taking advantage of the economy of scale. However, safe, inexpensive, and efficient methods to collect, store, and transport sputum samples from TB patients to these centralized facilities present some challenges.

Much of the world obtains a TB diagnosis using microscopic examination of Acid Fast Bacilli (AFB) stained sputum smears. Microscopy is also widely used for disease monitoring, even in regions that have adopted molecular testing for initial TB diagnoses [3]. AFB smears on glass slides are easily stored and shipped; they do not require temperature controlled storage and are not infectious. A number of studies have shown that DNA can be extracted from AFB smears and then used in TB DST analysis using molecular and other DNA sequencing based methods [5–12]. However, most of these studies involved small sample sizes (<100 slides) and only demonstrated the performance of their assays with one or two TB specific gene targets. Additionally, some studies used complex extraction and amplification protocols involving phenol-chloroform extractions, ethanol precipitations, and nested Polymerase Chain Reaction (PCR) amplifications requiring multiple primers for each target region, which would be impractical for widespread use.

We have developed a simple and effective method to extract *Mycobacterium tuberculosis* (MTB) DNA from clinically obtained AFB smears with sufficient quality for PCR amplification and next-generation DNA sequencing. We have also streamlined the process for PCR amplification of the isolated DNA for subsequent high-throughput sequencing of 17 regions of the *MTB* genome, enabling rapid detection of mutations associated with resistance to INH, RIF, ethambutol (EMB), PZA, FQ, and injectable aminoglycoside antibiotics. Here, we describe the method and assess the utility of our approach with 1,208 clinical AFB smears from Ghana, Kenya, Uganda, and Zambia. Since sequencing is becoming increasingly established as a definitive, reproducible, and reliable predictor of phenotypic DST, we also report the results of our new test desegregated by region and patient treatment status.

## Methods

### AFB direct sputum microscopy smears

**Fig 1:**
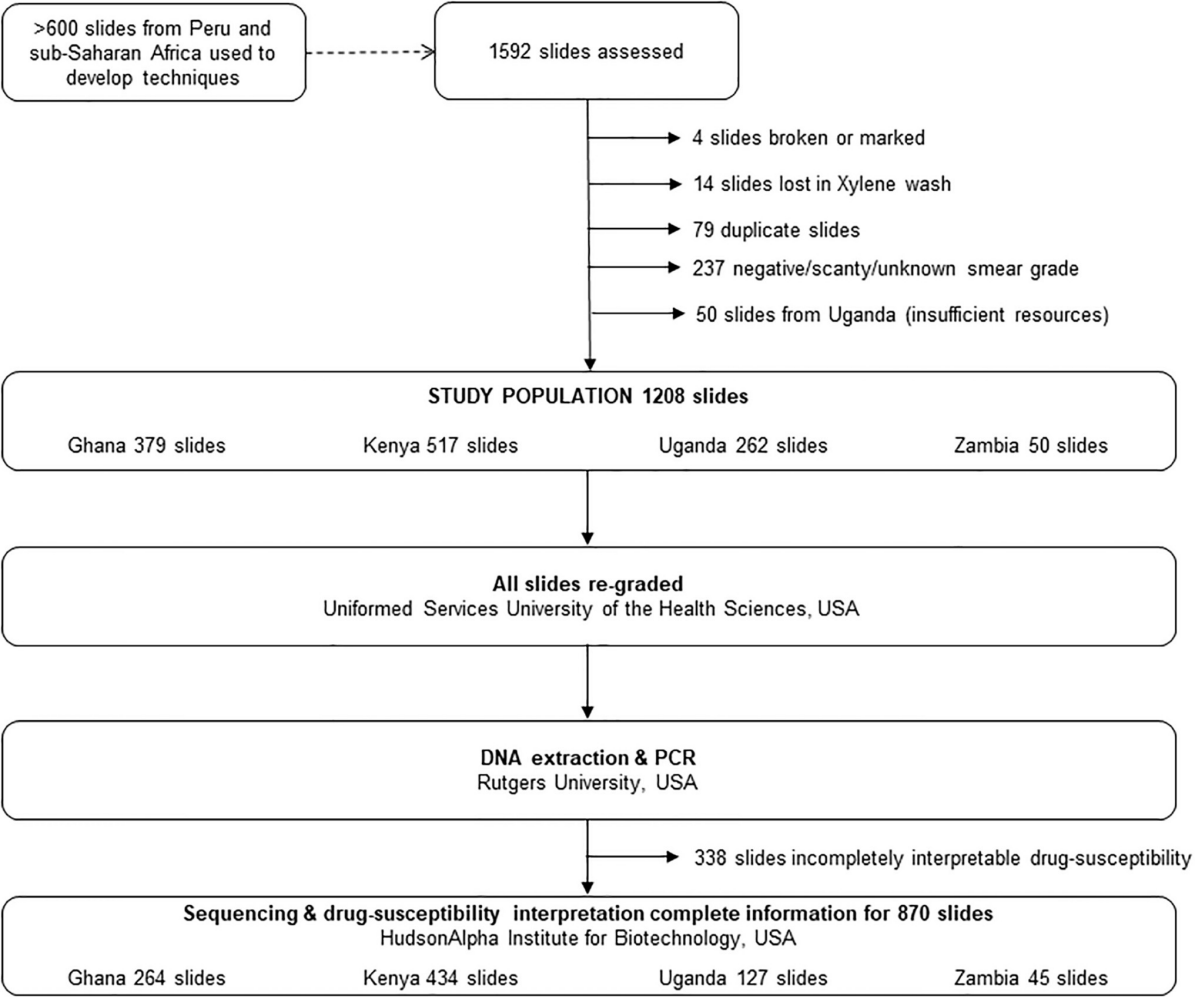
Flow diagram schematic of study sample population and methods.

A convenience sample of clinically obtained direct AFB sputum smears (n = 2,227), collected between 2013 and 2016, was provided for evaluation with our assay. We utilized 635 of the slides to develop our assay. We processed an additional 1,208 smears (one per subject) with the final version of our assay, which is the focus of this manuscript. A summary of the study sample population and methods is presented in Fig 1. We excluded 384 smears from processing (Fig 1). We excluded scanty [1 to 9 AFB in 100 fields] smears because a preliminary evaluation of our assay showed suboptimal performance with such smears (S1 Fig). The slides included in this study consisted of 379 smears from the Ghanaian Armed Forces Health Care Beneficiary population seen at 37 Military Hospital, Accra, 517 smears from a PEPFAR population in the Western highlands of Kenya, 262 smears from the Uganda Peoples Defence Force and Ministry of Health hospitals and the Central Public Health Laboratory, Uganda, and 50 smears from Maina Soko Military Hospital serving the greater Lusaka area of Zambia.

In each country, smears were prepared based on standard protocols recommended for clinical use by their national TB control programs. Types of stains used included Kinyoun, Auramine-Rhodamine, and Ziehl–Neelsen. All smears were centrally graded at the Uniformed Services University of the Health Sciences (USUHS) based on the International Union Against Tuberculosis and Lung Disease (IUATLD) grading system [13]; this required stripping and re-staining the Auramine-Rhodamine stained slides with Kinyoun stain (Remel, Lenexa, KS, USA). In the remainder of this manuscript, wherever a reference is made to stain type, we are specifically referring to the original stain type used on the smear.

### Ethics reviews

This study was reviewed and approved as non-human subjects research by the Institutional Review Boards (IRB) at the USUHS and Rutgers New Jersey Medical School, and it was reviewed and designated as an exempt protocol by the IRBs at the Naval Health Research Center, Walter Reed Army Institute of Research, Kenya Medical Research Institute, Makerere University, 37 Military Hospital, and the University of Zambia. Therefore, informed consent was waived by all aforementioned institutions. All data was anonymized before access by this study.

### Smear extraction and DNA isolation

Prior to extraction, the slides were washed with Xylene (Sigma Aldrich, Inc., Burlington, MA, USA) to remove immersion oil. For the extraction, 200 μl of Instagene Matrix (Bio-Rad Laboratories, Inc., Philadelphia, PA, USA) with 0.1% Triton X-100 (Sigma Aldrich, Inc., Burlington, MA, USA) was aliquoted into 1.5 mL Eppendorf tubes. Without disturbing the Chelex pellet, up to 100 μL of the Instagene Matrix and Triton X-100 solution was aspirated from the tube and dispensed onto the smear. The smear was then scraped off using a razor blade (Fisherbrand^™^ Razor Blades, Thermo Fisher Scientific, Inc., Hampton, NH, USA), aspirated off the slide, and transferred into the Eppendorf tube containing the Chelex pellet. To isolate the DNA, the extracted smear material was pulse vortexed at high speed for 30 seconds, then boiled for 20 minutes at 90°C, pulse vortexed on medium speed for 10 seconds, and centrifuged for 5 minutes at 15,000 rpm using Eppendorf Centrifuge 5424 (Eppendorf, Hamburg, Germany). The supernatant was transferred to a clean Eppendorf tube and purified using Qiagen QIAamp DNA Micro Kit (Qiagen, Inc., Germantown, MD, USA) according to manufacturer’s instructions for cleanup of genomic DNA. The purified DNA was eluted in 45 μL of AE elution buffer from the kit.

### Amplification targets and primers

The drug resistance phenotypes, gene target regions, and the associated segment names and primers are shown in Table 1. These target regions were selected to cover single nucleotide polymorphisms (SNPs) which have been reported to be associated with drug resistance in previous studies [14–16]. Mutation H57D in *pncA*, which occurs naturally in *M*. *bovis*, was excluded from downstream SNP analyses in this study. For *pncA*, the entire gene, including upstream and downstream flanking regions were targeted for amplification (Table 1).

**Table 1:** *Mycobacterium tuberculosis* gene regions targeted for detection of drug resistance associated mutations and associated primers.

Drug	Gene	Segment Name	Targeted Nucleotide Positions	Primer Direction	Primer Sequence (5’ to 3’)
Rifampicin	*rpoB*	*rpoB*	1215 to 1383	Forward	GATCACACCGCAGACGTTGA
				Reverse	ACGCTCACGTGACAGACCG
Isoniazid	*inhA promoter*	*inhA promoter*	nucleotide -160 to 44 of mabA gene	Forward	CCCAGAAAGGGATCCGTCAT
				Reverse	GATACGAATGGGGGTTTGGC
	*katG*	*katG*	809 to 992	Forward	CTGTTGTCCCATTTCGTCGG
				Reverse	GGCGGTCACACTTTCGGTAA
Ethambutol	*embB*	*emb10*	841 to 1017	Forward	GCCGTGGTGATATTCGGCTT
				Reverse	CAGCGCCAGCAGGTTGTAAT
		*emb20*	1156 to 1299	Forward	GCGGCGGCCATGGTCTTG
				Reverse	CAGCGCCGCCGGTGTGA
		*emb40*	1451 to 1550	Forward	CCGCCGGCACCGTCATCCTGA
				Reverse	GCCTGGCTCGGCCCGATTTTG
Fluoroquinolones/ Moxifloxacin/ Ofloxacin	*gyrA*	*gyrA*	159 to 395	Forward	CCGGGTGCTCTATGCAATGT
				Reverse	GCTTCGGTGTACCTCATCGC
Fluoroquinolones	*gyrB*	*gyrB*	1390 to 1639	Forward	GAGTTGGTGCGGCGTAAGAG
				Reverse	CCGTGATGATCGCCTGAACT
Kanamycin	*eis*	*eis promoter*	-81 to 34	Forward	CGTCCTCGGTCGGGCTACACAG
				Reverse	GCATCGCGTGATCCTTTGCCAGAC
Streptomycin	*rpsL*	*rpsL0*	54 to 170	Forward	GGTCAAGACCGCGGCTCTGA
				Reverse	AACTTCACGCGGGCAACCTTC
		*rpsL1*	183 to +53	Forward	CGAGGTCACGGCGTACATTC
				Reverse	GTAGACCGGGTCGTTGACCA
	*rrs*	*rrs10*	465 to 689	Forward	TCGGATTGACGGTAGGTGGA
				Reverse	CATTCCACCGCTACACCAGG
Streptomycin /Amikacin/Capreomycin/Kanamycin		*rrs30*	1368 to +59	Forward	ATACGTTCCCGGGCCTTGTA
				Reverse	AGACAAGAACCCCTCACGGC
Pyrazinamide	*pncA*	*pncA3*	-69 to 154	Forward	CAACAGTTCATCCCGGTTCG
				Reverse	TCGGTATTGCCACCGATCAT
		*pncA2*	111 to 337	Forward	CCAAGCCATTGCGTACCG
				Reverse	ATCCCAGTCTGGACACGTCG
		*pncA1*	245 to 454	Forward	CGTTCTCGTCGACTCCTTCG
				Reverse	AGCGGCGGACTACCATCAC
		*pncA0*	392 to +29	Forward	TGTGGAAGTCCTTGGTTGCC
				Reverse	CCCTATATCTGTGGCTGCCG

### Polymerase Chain Reactions (PCR)

All samples were amplified in 20 μL reactions. Smears graded 1+ were amplified in uniplex reactions containing 2 μl of target DNA to increase PCR efficacy and sequencing depth for these paucibacillary samples. For smears graded 2+ or 3+ all gene target sequences were amplified in duplexed reactions using 3 or 4 μL of target DNA per reaction, except for *gyrB*, which was only amplified in a uniplex reaction to maintain amplification efficiency. The PCR mix consisted of the following: 2.5 mM magnesium chloride (MgCl_2_)), 0.25 mM deoxyribonucleotides, 5% glycerol, 1X PCR buffer without MgCl_2_, 1 unit Jumpstart Taq DNA polymerase, and 0.5 μM primers (all reagents from Sigma Aldrich, Inc., Burlington, MA, USA). Samples were PCR-amplified in Roche LightCycler^®^ 480 System (Roche Molecular Systems, Inc., Branchburg, NJ, USA) and Applied Biosystems Prism 7900HT Fast Real-Time PCR System (Applied Biosystems, Foster City, USA). For duplex reactions, primers were paired as follows: *rpoB-pncA3*, *rrs30-rpsL1*, *emb10-katG*, *emb20-eis*, *emb40-rpsL0*, *inhA-pncA2*, *gyrA-pncA0*, and *rrs10-pncA1*. All PCR products for each sample were pooled post amplification. For amplification, samples were denatured at 95°C for 1 minute followed by 40 cycles of touchdown PCR with the following parameters: 1) 10 cycles with 95°C denaturation for 15 seconds, annealing for 15 seconds starting at 70°C then lowered by 1°C with each subsequent cycle, and extension at 72°C for 30 seconds; 2) 30 cycles with 95°C denaturation for 15 seconds, annealing at 60°C for 15 seconds, and extension at 72°C for 30 seconds. With each PCR batch, a negative (dH_2_O) control, a wild type positive control (H37Rv genomic DNA), and genomic DNA extracted from a mutant positive control drug-resistant strain (TB-TDR-0114 or TB-TDR-0115)) were included [17]. These controls were also included in the downstream sequencing and SNP detection pipeline.

### Sequencing

The samples were sequenced at the HudsonAlpha GSL (Huntsville, AL, USA) using an Ilumina MiSeq^™^ platform (Illumina, Inc., San Diego, CA, USA) (S1 Appendix). Sequencing results have been deposited in the National Center for Biotechnology Information (NCBI) Sequence Read Archive (SRA) (BioProject numbers: PRJNA608715, PRJNA608724).

### Data analysis

Reads were aligned to a reference sequence from the H37Rv strain of TB using an in-house bioinformatics pipeline (S2 Appendix). For each DST target region of a given slide, results were classified as interpretable if coverage depth of 20X or more reads was obtained; a mutation frequency of 80% or greater was considered sufficient for calling SNPs. If coverage depth of a DST target region was less than 20X, the results for that target region were classified as un-interpretable. Prevalence and corresponding exact binomial confidence intervals of mutations associated with resistance to first- and second-line anti-tuberculosis drugs were estimated for all slides with interpretable results for all gene targets.

### Results

Slide characteristics are summarized by country of origin in Table 2. The 1,208 processed slides consisted of 282 (23.1%) 1+ smears, 352 (29.1%) 2+ smears, and 574 (47.5%) 3+ smears. All Kenya smears and 58% of Ghana smears were from adult patients. Information on adult or child status was missing for Uganda and Zambia smears. Over 61% of the smears were classified as new TB cases. The majority of the smears (42.8%) were from Kenya (Table 2). Ziehl-Neelsen was the most frequently used stain (Table 2).

**Table 2:** Sample characteristics by country.

Sample Characteristics	Ghana (%)	Kenya (%)	Uganda (%)	Zambia (%)	Total (%)
**Sample Size**	379 (31.4)	517 (42.8)	262 (21.7)	50 (4.1)	1208 (100)
**Smear Grade**					
1+	72 (19.0)	174 (33.7)	21 (8.0)	15 (30.0)	282 (23.3)
2+	62 (16.4)	141 (27.3)	131 (50.0)	18 (36.0)	352 (29.1)
3+	245 (64.6)	202 (39.1)	110 (42.0)	17 (34.0)	574 (47.5)
**Stain Type**^a^					
Ziehl Neelsen	379 (100)	0	49 (18.7)	0	428 (35.4)
Kinyoun	0	341 (66.0)	0	50 (100)	391 (32.4)
Auramine/Rhodamine	0	176 (34.0)	207 (79.0)	0	383 (31.7)
Unknown	0	0	6 (2.3)	0	6 (0.5)
**Treatment Status**					
New case	172 (45.4)	352 (68.1)	221 (84.4)	0	745 (61.7)
Follow Up	0	124 (24.0)	35 (13.4)	0	158 (13.1)
Retreatment	0	38 (7.4)	4 (1.5)	0	43 (3.6)
Unknown	207 (54.6)	3 (0.6)	2 (0.8)	50 (100)	262 (21.7)

^a^Stain type specified in this table is the original type of stain used on the smear at the clinical site where sample was collected.

**Fig 2:**
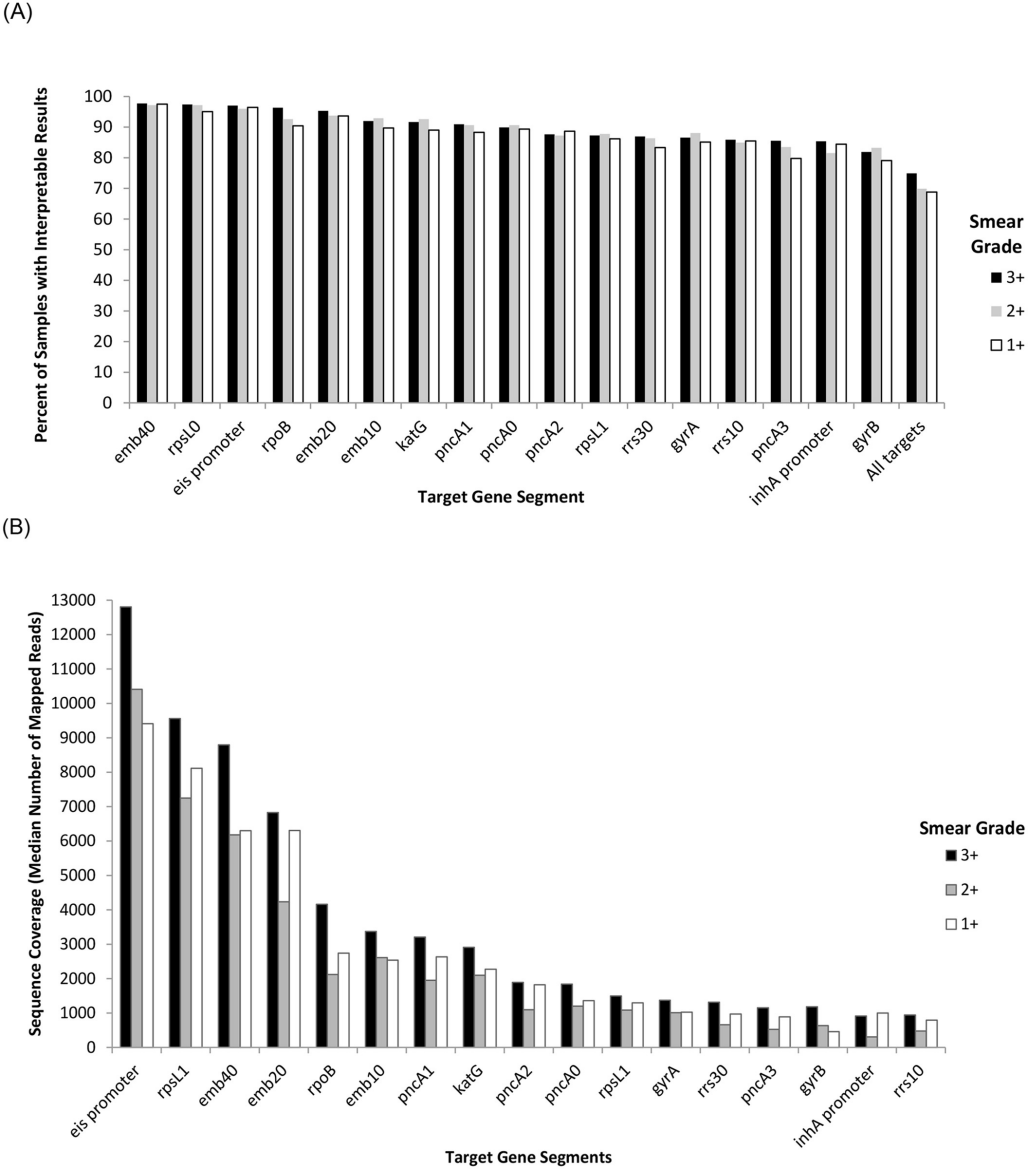
Percent of samples per smear grade with interpretable results and median number of mapped reads per target gene segment. A) Percent of samples per smear grade with interpretable results (coverage depth of 20X or greater) for all and specified target gene segments; B) Sequence coverage in median number of mapped reads per target gene segment, stratified by smear grade.

Coverage depth varied by target region. The percentage of slides per smear grade with interpretable results overall and per target gene segments is reported in Fig 2A. With the exception of gyrB (interpretable results obtained in 79% of 1+ smears), interpretable results were obtained for any given target gene segment in 80% or more of slides, regardless of smear grade (Fig 2A). Interpretable results was obtained for all targets in 194 (68.8%) 1+ smears, 246 (69.9%) 2+ smears, 430 (74.9%) 3+ smears, and 870 (72%) slides overall. The number of mapped reads varied by target gene segment and slide smear grade, ranging from approximately 300X for *rrs10* to over 12000X for the *eis* promoter on average. Median coverage per target region, stratified by smear grade, is shown in Fig 2B. Coverage depth also varied by stain type. Interpretable results were obtained for all targeted gene segments in over 94% of Kinyoun stained smears, but only 64% of Ziehl-Neelsen stained smears, and 59% of Auramine/Rhodamine stained smears (S1 Table).

A summary of all drug resistance associated mutations detected in all gene targets are presented in Table 3. RIF resistance associated mutations in *rpoB* were detected in 6.3% of new cases, 3.3% of follow-up cases (patients under initial treatment), 20.0% of retreatment cases (S2 Table), and 6.1% of slides overall (Table 3). The highest rate of RIF resistance associated mutations were detected in slides from Uganda (18.3%), followed by slides from Zambia (8.51%). Over 85% of RIF resistance associated mutations occurred in codon 445 (alternative numbering system: 526) of *rpoB* (Table 3).

**Table 3:** Summary of drug resistance associated mutations detected in direct AFB smears and total number of drug resistant smears from Ghana, Kenya, Uganda, and Zambia, by drug type^a^.

Drug	Gene Segment	Nucleotide (Reference/Mutant)	Amino Acid Alteration^b^	Ghana	Kenya	Uganda	Zambia	Total
Rifampicin	*rpoB*	1289 CTG/ CCG	L430P (L511P)	0/345	0/496	2/246	0/47	2/1134
		1295 CAA/ CCA	Q432P (Q513P)	1/345	0/496	0/246	0/47	1/1134
		1304 GAC/ GTC	D435V (D516V)	0/345	1/496	0/246	1/47	2/1134
		1333 CAC/GAC	H445D (H526D)	1/345	0/496	41/246	3/47	45/1134
		1333 CAC/TAC	H445Y (H526Y)	2/345	10/496	0/246	0/47	12/1134
		1333 CAC/AAC	H445N (H526N)	1/345	0/496	0/246	0/47	1/1134
		1349 TCG/ TTG	S450L (S531L)	2/345	0/496	2/246	0/47	4/1134
		1355 CTG/ CCG	L452P (L533P)	2/345	0/496	0/246	0/47	2/1134
**Total Rifampicin Resistant Smears (%)**				**9/345 (2.61)**	**11/496 (2.22)**	**45/246 (18.29)**	**4/47 (8.51)**	**69/1134 (6.08)**
Isoniazid	*inhA Promoter*	-15 C/T	Promoter	3/326	0/476	2/166	0/47	5/1015
*katG*	944 AGC/ACC	S315T	24/337	14/501	16/218	6/47	60/1103
**Total Isoniazid Resistant Smears (%)**				**27/316 (8.54)**	**14/473 (2.96)**	**18/168 (10.71)**	**6/47 (12.77)**	**65/1004 (6.47)**
Streptomycin	*rpsL0*	128 AAG/AGG	K43R	14/365	13/512	0/242	0/50	27/1169
	*rpsL1*	263 AAG/AGG	K88R	5/312	0/490	3/204	0/47	8/1053
	*rrs10*	513 A/C		0/319	3/480	0/186	0/48	3/1033
	*rrs10*	516 C/T		0/319	0/480	2/186	0/48	2/1033
**Total Streptomycin Resistant Smears (%)**				**19/292 (6.51)**	**16/468 (3.42)**	**5/179 (2.79)**	**0/47**	**40/986 (4.06)**
Ethambutol	*embB10*	916 ATG/GTG	M306V	2/343	13/500	4/218	1/47	20/1108
	*embB10*	918 ATG/ATA	M306I	1/343	0/500	2/218	0/47	3/1108
	*embB20*	1190 GGC/GAC	G406D	0/360	0/505	3/228	1/47	4/1140
**Total Ethambutol Resistant Smears (%)**				**3/339 (0.88)**	**13/496 (2.62)**	**7/218 (3.21)**^**c**^	**2/47 (4.26)**	**25/1100 (2.27)**^**c**^
Fluoroquinolones	*gyrA*	281 GAC/GGC	D94G	2/314	12/486	0/200	0/47	14/1047
**Total Fluoroquinolones Resistant Smears (%)**^**g**^				**2/281 (0.71)**	**12/459 (2.61)**	**0/180**	**0/46**	**14/966 (1.45)**
Moxifloxacin/ Ofloxacin	*gyrA*	281 GAC/GGC	D94G	2/314	12/486	0/200	0/47	14/1047
**Total Moxifloxacin/ Ofloxacin Resistant Smears (%)**				**2/314 (0.64)**	**12/486 (2.47)**	**0/200**	**0/47**	**14/1047 (1.34)**
Pyrazinamide	*pncA3*	-11 A/G	Promoter	0/319	10/475	0/170	0/46	10/1010
	*pncA3*	11 TTG/TCG	L4S	0/319	1/475	0/170	0/46	1/1010
	*pncA3*	35 GAC/GCC	D12A	0/319	0/475	1/170	0/46	1/1010
	*pncA3*	37 TTC/CTC	F13L	1/319	0/475	0/170	0/46	1/1010
	*pncA3*	104 CTG/CGG	L35R	0/319	0/475	0/170	2/46	2/1010
	*pncA3*	137 GCA/GTA	A46V	1/319	0/475	0/170	0/46	1/1010
	*pncA2*	151 CAC/TAC	H51Y	1/327	0/487	0/199	0/47	1/1060
	*pncA2*	185 CCG/CAG; 185 CCG/CTG	P62Q; P62L Mixture	1/327	0/487	0/199	0/47	1/1060
	*pncA2*	188 GAC/GGC	D63G	0/327	0/487	2/199	0/47	2/1060
	*pncA2*	202 TGG/CGG	W68R	1/327	0/487	0/199	0/47	1/1060
	*pncA1*	322 GGA/AGA	G108R	1/335	0/496	0/211	0/48	1/1090
**Total Pyrazinamide Resistant Smears (%)**				**6/302 (1.99)**	**11/467 (2.36)**	**3/160 (1.88)**	**2/46 (4.35)**	**22/975 (2.26)**

^a^ A coverage depth cut-off of 20X and a frequency cut-off of 80% was used for all reported single nucleotide polymorphisms. For each target gene segment, the number of smears with SNPs out of the total number of smears with 20X or greater coverage for the given target gene segment are reported per country. For each country and in total, the percent of smears with resistance to a given drug were calculated by dividing the number of smears with at least one mutation associated with resistance to the given drug by the total number of smears in which all relevant target gene segments (listed in Table 1) were successfully screened plus any smears in which at least one associated target gene segment met the coverage cut-off and contained a drug resistance associated mutation.

^b^ For rpoB0, the amino acid number is presented in the MTB numbering system followed by the alternative numbering system in parentheses.

^c^ Two samples from Uganda contained a drug resistant associated mutation in both *emb10* and *emb20* gene segments. The numerator has been adjusted to ensure we do not double count these samples in calculating percent of etambutol resistant smears from Uganda and overall.

INH resistance associated mutations in *katG* and/or *inhA* were detected in 6% of slides overall, with the highest rate being among Zambia slides (12.8%), followed by Uganda (10.7%) (Table 3). Most (92%) of the observed INH resistance associated mutations were S315T in *katG* (Table 3). Overall, fluoroquinolones, moxifloxacin, and ofloxacin resistance associated mutations in *gyrA* were observed in over 1% of slides (Table 3). PZA resistance associated mutations in *pncA* were observed in over 2% of slides overall (Table 3). Majority of the distinct *pncA* mutations were only detected in a single slide. However, the -11 A/G mutation in the *pncA* promoter was observed in 2.4% of Kenya slides. Detected *pncA* SNPs for which there currently is little to no evidence of an association with drug resistance are reported in S3 Table. We did not detect any SNPs in our wild-type positive controls or negative controls.

Prevalence estimates and associated 95% confidence intervals for detected drug resistance mutation profiles in smears with interpretable results for all DST gene targets, stratified by country are presented in Table 4. Overall, drug resistance associated SNPs were detected in 88/870 (Estimate: 10.11%; 95% CI: 8.19%, 12.31%) of the slides with interpretable results for all DST gene targets, with mutation rates being highest in slides from Zambia (Table 4). Approximately 58% of the slides in which a drug resistance associated mutation was detected contained a SNP associated with mono-resistance to RIF, INH, or SM, with the most frequent mutation being associated with INH resistance (Table 4). A MDR mutation profile with resistance to at least RIF and INH was also observed in 8% (95% CI: 2.48%, 21.22%) of smears from Zambia and in 1.8% (95% CI: 1.05%, 2.97%) of smears overall (Table 4). No XDR mutation profiles were identified.

**Table 4:** Prevalence and 95% confidence intervals for detected drug resistance mutation profiles in smears with interpretable results for all target gene segments, stratified by country^a^.

Mutation Based Drug Resistance Profile	Ghana		Kenya		Uganda		Zambia		Total	
	Prevalence (%)	95% CI	Prevalence (%)	95% CI	Prevalence (%)	95% CI	Prevalence (%)	95% CI	Prevalence (%)	95% CI
**Mono-resistance**										
RIF only	1/264 (0.38)	0.01, 2.09	10/434 (2.30)	1.11, 4.20	6/127 (4.72)	1.75, 10.00	0/45 (0.00)	0.00, 7.87	17/870 (1.95)	1.14, 3.11
INH only	14/264 (5.30)	2.93, 8.74	0/434	0.00, 0.85	6/127 (4.72)	1.75, 10.00	2/45 (4.44)	0.54, 15.15	22/870 (2.53)	1.59, 3.80
SM only	9/264 (3.41)	1.57, 6.37	2/434 (0.46)	0.06, 1.66	0/127 (0.00)	0.00, 2.86	0/45 (0.00)	0.00, 7.87	11/870 (1.26)	0.63, 2.25
PZA only	0/264 (0.00)	0.00, 1.39	0/434 (0.00)	0.00, 0.85	0/127 (0.00)	0.00, 2.86	1/45 (2.22)	0.06, 11.77	1/870 (0.11)	0.00, 0.64
**Multi-drug Resistance**										
RIF and INH	4/264 (1.52)	0.41, 3.83	0/434 (0.00)	0.00, 0.85	1/127 (0.79)	0.02, 4.31	3/45 (6.67)	1.40, 18.27	8/870 (0.92)	0.40, 1.80
RIF, INH, and one or more of the following: EMB, SM, PZA	3/264 (1.14)	0.23, 3.28	1/434 (0.23)	0.01, 1.28	3/127 (2.36)	0.49, 6.75	1/45 (2.22)	0.06, 11.77	8/870 (0.92)	0.40, 1.80
**Poly-resistance**										
Two or more of the following: INH, SM, EMB, PZA	6/264 (2.27)	0.84, 4.88	0/434 (0.00)	0.00, 0.85	3/127 (2.36)	0.49, 6.75	1/45 (2.22)	0.06, 11.77	10/870 (1.15)	0.55, 2.10
FQ, MXF, and OFL	1/264 (0.38)	0.01, 2.09	0/434 (0.00)	0.00, 0.85	0/127 (0.00)	0.00, 2.86	0/45 (0.00)	0.00, 7.87	1/870 (0.11)	0.00, 0.64
INH, EMB, SM, PZA, FQ, MXF, OFL	0/264 (0.00)	0.00, 1.39	10/434 (2.30)	1.11, 4.20	0/127 (0.00)	0.00, 2.86	0/45 (0.00)	0.00, 7.87	10/870 (1.15)	0.55, 2.10
**Total**	**38/264 (14.39)**	**10.39, 19.22**	**23/434 (5.30)**	**3.99, 7.85**	**19/127 (14.96)**	**9.25, 22.37**	**8/45 (17.78)**	**8.00, 32.05**	**88/870 (10.11)**	**8.19, 12.31**

^a^Abbreviations: RIF = rifampicin; INH = isoniazid; SM = streptomycin; PZA = pyrazinamide; EMB = ethambutol; FQ = fluoroquinolone; MXF = moxifloxacin; OFL = ofloxacin; CI = confidence interval.

Presented in Table 5 are overall and country specific prevalence estimates and 95% confidence intervals for phenotypic interpretation of observed mutations in smears with interpretable results for all DST gene targets, stratified by case treatment status. Among smears with interpretable results for all gene targets, 7.8% of new cases, 4.1% of follow up cases, and 28.1% of retreatment cases had mutation profiles for mono-resistance, MDR, or poly-resistance.

**Table 5:** Overall and country specific prevalence and 95% confidence intervals for phenotypic interpretation of observed mutations in smears with interpretable results for all gene targets, by case treatment status.

Case Treatment Status by Country	No Drug Resistance		Mono-Resistance		Multi-Drug Resistance		Poly-Resistance		Any Drug Resistant Smears	
	Prevalence (%)	95% CI	Prevalence (%)	95% CI	Prevalence (%)	95% CI	Prevalence (%)	95% CI	Prevalence (%)	95% CI
**Ghana**										
New	111/125 (88.80)	81.92, 93.74	13/125 (10.40)	5.65, 17.13	1/125 (0.80)	0.02, 4.38	0/125 (0.00)	0.00, 2.91	14/125 (11.20)	6.26, 18.08
Unknown	115/139 (82.73)	75.41, 85.61	11/139 (7.91)	4.02, 13.72	6/139 (4.32)	1.60, 9.16	7/139 (5.04)	2.05, 10.10	24/139 (17.27)	11.39, 24.59
**Total**	226/264 (85.61)	80.78, 89.61	24/264 (9.09)	5.91, 13.22	7/264 (2.65)	1.07, 5.39	7/264 (2.65)	1.07, 5.39	38/264 (14.39)	10.39, 19.22
**Kenya**										
New	287/297 (96.63)	93.90, 98.37	4/297 (1.35)	0.37, 3.41	1/297(0.34)	0.01, 1.86	5/297 (1.68)	0.55, 3.88	10/297 (3.37)	1.63, 6.10
Follow-up	100/104 (96.15)	90.44, 98.94	1/104 (0.96)	0.02, 5.10	0/104 (0.00)	0.00, 3.48	3/104 (2.88)	0.60, 8.20	4/104 (3.85)	1.06, 9.56
Retreatment	23/31 (74.19)	55.39, 88.14	7/31 (22.58)	9.59, 41.10	0/31 (0.00)	0.00, 11.22	1/31 (3.23)	0.08, 16.70	8/31 (25.81)	11.86, 44.61
Unknown	1/2 (50.00)	1.26, 98.74	0/2 (0.00)	0.00, 84.19	0/2 (0.00)	0.00, 84.19	1/2 (50.00)	1.26, 98.74	1/2 (50.00)	1.26, 98.74
**Total**	411/434 (94.70)	92.15, 96.61	12/434 (2.76)	1.44, 4.78	1/434 (0.23)	0.01, 1.28	10/434 (2.30)	1.11, 4.20	23/434 (5.30)	3.39, 7.85
**Uganda**										
New	90/107 (84.11)	75.78, 90.46	12/107 (11.21)	5.93, 18.77	3/107 (2.80)	0.58, 7.98	2/107 (1.87)	0.23, 6.59	17/107 (15.89)	9.54, 24.22
Follow-up	17/18 (94.44)	72.71, 99.86	0/18 (0.00)	0.00, 18.53	1/18 (5.56)	0.14, 27.29	0/18 (0.00)	0.00, 18.53	1/18 (5.56)	0.14, 27.29
Retreatment	0/1 (0.00)	0.00, 97.50	0/1 (0.00)	0.00, 97.50	0/1(0.00)	0.00, 97.50	1/1 (100.00)	2.50, 100.00	1/1 (100.00)	2.50, 100.00
Unknown	1/1 (100)	2.50, 100.00	0/1 (0.00)	0.00, 97.50	0/1(0.00)	0.00, 97.50	0/1 (0.00)	0.00, 97.50	0/1(0.00)	0.00, 97.50
**Total**	108/127 (85.04)	77.63, 90.75	12/127 (9.45)	4.98, 15.92	4/127 (3.15)	0.86, 7.87	3/127 (2.36)	0.49, 6.75	19/127 (14.96)	9.25, 22.37
**Zambia**										
Unknown	37/45 (82.22)	67.95, 92.00	3/45 (6.67)	1.40, 18.27	4/45 (8.89)	2.48, 21.22	1/45 (2.22)	0.06, 11.77	8/45 (17.78)	8.00, 32.05
**Total**	37/45 (82.22)	67.95, 92.00	3/45 (6.67)	1.40, 18.27	4/45 (8.89)	2.48, 21.22	1/45 (2.22)	0.06, 11.77	8/45 (17.78)	8.00, 32.05
**Overall**										
New	488/529 (92.25)	89.63, 94.38	29/529 (5.48)	3.70, 7.78	5/529 (0.95)	0.31, 2.19	7/529 (1.32)	0.53, 2.71	41/529 (7.75)	5.62, 10.37
Follow-up	117/122 (95.90)	90.69, 98.66	1/122 (0.82)	0.02, 4.48	1/122 (0.82)	0.02, 4.48	3/122 (2.46)	0.51, 7.02	5/122 (4.10)	1.34, 9.31
Retreatment	23/32 (71.88)	53.25, 86.25	7/32 (21.88)	9.28, 39.97	0/32 (0.00)	0.00, 10.89	2/32 (6.25)	0.77, 20.81	9/32 (28.13)	13.75, 46.75
Unknown	153/187 (81.82)	75.53, 87.07	14/187 (7.49)	4.15, 12.24	10/187 (5.35)	2.59, 9.61	9/187 (4.81)	2.22, 8.94	33/187 (17.65)	12.47, 23.88
**Total**	**782/870 (89.89)**	**87.69, 91.81**	**51/870 (5.86)**	**4.40, 7.64**	**16/870 (1.84)**	**1.05, 2.97**	**21/870 (2.41)**	**1.50, 3.67**	**88/870 (10.11)**	**8.19, 12.31**

We re-sequenced a subset of the slides using Sanger sequencing to confirm the accuracy of our MiSeq based approach. This included 16 slides with a wild type *pncA* gene, 61 with *rpoB* mutations, 18 with *KatG* mutations, and 27 additional slides with mutations in other gene targets. Our Sanger sequencing results matched the results of our primary study in every case except for one slide where an H445D mutation was detected in *rpoB* by MiSeq (coverage depth: 1700X; mutation frequency: 99%) but not Sanger sequencing (S1, S2 and S3 Data).

## Discussion

We have demonstrated that DNA of sufficient quality for PCR amplification and next generation DNA sequencing can be isolated from AFB stained direct sputum smears and tested for mutations associated with resistance to all first and several second line anti-tuberculosis drugs. The DNA isolation method we have developed is simple and rapid and does not require much technical expertise. The samples used in this study were comprised of a diverse set of clinically obtained smears in terms of smear grade, AFB stain type, and geographic origin. Although the percentage of slides that met the coverage cut-off of 20X was similar for all target gene segments, the median number of reads for each target varied. This indicates that while sufficient DNA can be isolated from 1+ to 3+ smears to amplify and sequence all gene segments tested in this study, certain targets (e.g., *eis* promoter) are amplified more efficiently than others by the selected primer pairs. Kinyoun stained smears enabled better sequencing coverage compared to other stain types. However, the poor performance with Auramine/Rhodamine stained smears could in part be due to subsequent stripping and counterstaining of these slides with Kinyoun stain at USUHS, which may have resulted in DNA loss. Given that each country mainly used one type of stain, the variation observed in coverage depth by stain type could also be due to site-specific factors (Table 2).

Our assay demonstrated well-established associations of drug-resistance with geographical region and TB treatment status. Although the number of retreatment cases in our study was small, the rate of RIF resistance associated mutations we observed in retreatment cases as compared to new cases was similar to trends currently reported for the continent of Africa, where the rate of drug resistance mutations in retreatment cases is almost four times that observed in new TB cases [3]. The countries from which slides were analyzed in our study are on the WHO’s list of top high burden countries for TB (Kenya; Zambia), MDR-TB (Kenya), and/or TB-HIV concurrent infections (Zambia; Ghana; Uganda) [3]. Comprehensive genotypic drug resistance surveillance data for these countries is currently limited. Based on national surveillance data, less than 2% of new TB cases are drug-resistant in all countries from which slides were included in our study [3]. Although our sample size for each country was small and subject treatment status was only known for a subset of our samples, the estimated prevalence of drug resistance among new TB cases in our study was over ten times higher than reported national rates for Ghana and Uganda. Among Kenyan smears in our sample, the estimated drug resistance rate was almost three times higher than the reported national rate. It could be that a greater number of drug-resistant cases were evaluated at the facilities that we received our samples from because they are centralized laboratories. The drug resistance rate estimated among Zambian smears in our sample was similar to the national rate of 18% reported for relapse cases in Zambia [3]. However, the subject treatment status associated with the Zambian slides in our study was unknown. Similar to findings from previous phenotypic and molecular drug resistance surveillance studies [18–26], we found INH mono-resistance associated mutations to be more common in samples overall than RIF mono-resistance or RIF-INH dual resistance. Noted is that Ghana likely has both *M*. *africanum* and *M*. *tuberculosis* infection in clinically identified TB subjects, cited as up to 40% in West Africa [24]. This may be relevant to our study, as *M*. *africanum* has been reported to have less drug resistance compared to *M*. *tuberculosis* in Ghana [25]. Differentiation between *M*. *africanum* and *M*. *tuberculosis* was not possible with the target regions we sequenced.

A limitation of this study is that our specimens were obtained from central or regional reference laboratories and thus may not be generalizable to the entire population. Another limitation is that the observed drug resistance mutations could not be confirmed via phenotypic DST, given that the study was conducted retrospectively. Nevertheless, we were able to validate the in-house bioinformatics pipeline that was used to analyze our samples by reanalyzing the data for 384 slides with the previously published ASAP pipeline [26]. We observed no discordance between the variant calls made by our pipeline and the ASAP pipeline when adjusting coverage cut-off to 20X and SNP frequency to 80% (S3 Appendix). Also, limiting our screening process only to previously published drug-resistant mutations may have led to an underestimation of drug resistance in our samples.

Among the slides processed by Sanger and MiSeq sequencing, one discordant sample was observed, where *rpoB* mutation H445D (alternate naming system: H526D) was detected by MiSeq but not by Sanger sequencing. Given the length of the *rpoB* target, coverage of codon 445 was only available from the forward direction from Sanger sequencing. It is possible that the *rpoB* SNP was missed by Sanger sequencing in this sample. Alternatively, the portion of the discordant sample amplified by MiSeq sequencing may have been contaminated. Regardless, the overall concordance between the two approaches was very high. Although precautions were taken to prevent contamination, some samples could have been contaminated during sample processing.

Our approach has several potential advantages compared to phenotypic DSTs and to currently available rapid molecular DST tests. Our approach is free from the biohazards associated with phenotypic DST and can be performed without use of expensive biocontainment laboratories. Compared to commercially available rapid molecular tests, our use of targeted DNA sequencing enables easy expansion to detect additional resistance mutations as well as mutations to new drugs as needed. The total reagent costs for our approach, including all assay steps, was approximately $50 per slide, for slides amplified with duplex PCR, and $60 for slides amplified with uniplex PCR. However, with additional multiplexing of the PCR step and with expected decreases in next generation sequencing costs, it is likely that our approach could soon become less expensive than other DST methods. Aside from the smear scraping step, slides can be batch processed at every step of the process. Slide scraping to DNA purification can be completed for at least 12 samples in less than an hour.

In summary, we have demonstrated a simple and rapid method for determining drug resistance in a widely available sample type, AFB stained sputum microscopy smears, which is easily storable, and safely transportable without infectious risk. This approach should prove useful for diagnosing drug-resistant TB as well as for surveillance purposes.

Future research may add mycobacterial speciation targets, which may determine the frequency of positive acid-fast microscopy caused by mycobacteria other than MTB.

## Supporting information

S1 Appendix HudsonAlpha sequencing protocol. (DOCX)

S2 Appendix Description of bioinformatics pipeline. (DOCX)

S3 Appendix Links to output generated by the ASAP pipeline. (DOCX)

S1 Fig Percent of scanty smears with interpretable results overall and per target gene segment (A) and median number of mapped reads per target gene segment for scanty smears (B). (DOCX)

S1 Table Number and percent of smears with interpretable results for all target gene segments by stain type, overall and per smear grade. (DOCX)

S2 Table Summary of drug resistance associated mutations detected in direct AFB smears by case status and drug type. (DOCX)

S3 Table Summary of non-drug resistance associated mutations detected in pncA gene of DNA isolated from direct AFB smears from Ghana, Kenya, Uganda, and Zambia. (DOCX)

S1 Data (ZIP)

S2 Data (ZIP)

S3 Data (XLSX)
