# Supplementary material for: Detection of drug resistant Mycobacterium tuberculosis by high-throughput sequencing of DNA isolated from acid fast bacilli smears
Source: PLoS One. 2020 May 8;15(5):e0232343. doi: 10.1371/journal.pone.0232343 (PMC7209238; doi:10.1371/journal.pone.0232343)
Supplement: S1 Appendix — (DOCX) [file pone.0232343.s001.docx]

**S1 Appendix: HudsonAlpha sequencing protocol.**

Approximately, 10 uL of DNA from each sample was put through 8 cycles of PCR using KAPA HiFi HotStart Ready Mix (Kapa Biosystems, Inc., Woburn, MA, USA) and dual custom GSL index primers. The concentration of the libraries was assessed by Picogreen. Each sample was normalized and pooled equally and the pool was purified. The fragment size of the final pool was assessed using the Agilent Bioanalyzer (Aligent, Santa Clara, CA, USA). Accurate quantification for sequencing applications for the pool was determined using the qPCR-based KAPA Biosystems Library Quantification kit (Kapa Biosystems, Inc., Woburn, MA, USA). The pool was sequenced on the MiSeq platform using v1 kit flow cells and reagents at 150 bp paired-end (PE) sequencing.
