# Supplementary material for: Detection of drug resistant Mycobacterium tuberculosis by high-throughput sequencing of DNA isolated from acid fast bacilli smears
Source: PLoS One. 2020 May 8;15(5):e0232343. doi: 10.1371/journal.pone.0232343 (PMC7209238; doi:10.1371/journal.pone.0232343)
Supplement: S2 Appendix — (DOCX) [file pone.0232343.s002.docx]

**S2 Appendix: Description of bioinformatics pipeline.**

AlienTrimmer version 0.4.0 was used to remove adaptor sequences and low-quality read ends. An in-house script was used to filter out unpaired reads due to trimming. BWA version 0.7.9a-r786 was used to align reads to the reference file with the commands "bwa aln" and "bwa sampe". Variants were called with Samtools version 0.1.17 with the command "samtool -uf mpileup" followed by bcftools version 0.1.17 (r973:277). The number of mapped reads per base on the reference was calculated with bedtools version 2.17.0 with the command "bedtools genomecov".
