# Supplementary material for: Detection of drug resistant Mycobacterium tuberculosis by high-throughput sequencing of DNA isolated from acid fast bacilli smears
Source: PLoS One. 2020 May 8;15(5):e0232343. doi: 10.1371/journal.pone.0232343 (PMC7209238; doi:10.1371/journal.pone.0232343)
Supplement: S3 Appendix — (DOCX) [file pone.0232343.s003.docx]

**S3 Appendix: Links to output generated by the ASAP pipeline.**

**Link1** (samples compared to the ASAP assay reference sequences for TB gene targets):

https://pathogen.tgen.org/ASAP/TB_AFB_ASAP_test1.html

**Link2** (samples compared to the reference sequences used in our study):

https://pathogen.tgen.org/ASAP/TB_AFB_ASAP_test2.html
