# Supplementary material for: Detection of drug resistant Mycobacterium tuberculosis by high-throughput sequencing of DNA isolated from acid fast bacilli smears
Source: PLoS One. 2020 May 8;15(5):e0232343. doi: 10.1371/journal.pone.0232343 (PMC7209238; doi:10.1371/journal.pone.0232343)
Supplement: S1 Fig — (DOCX) [file pone.0232343.s004.docx]

(A)

(B)

S1 Figure. Percent of samples with interpretable results and sequence coverage depth per target gene segment for scanty smears. A) Percent of scanty smears (n=6) with interpretable results per target gene segment and all targets. Interpretable result is defined as coverage depth of 20X or greater. B) Sequence coverage depth (median number of mapped reads) per target gene segment for scanty smears (n=6).
