# Supplementary material for: Detection of drug resistant Mycobacterium tuberculosis by high-throughput sequencing of DNA isolated from acid fast bacilli smears
Source: PLoS One. 2020 May 8;15(5):e0232343. doi: 10.1371/journal.pone.0232343 (PMC7209238; doi:10.1371/journal.pone.0232343)
Supplement: S1 Table — (DOCX) [file pone.0232343.s005.docx]

| S1 Table. Number and percent of smears with interpretable results for all target gene segments by stain type, overall and per smear grade.* | | | |  |  |
| --- | --- | --- | --- | --- | --- |
| **Stain type** | **Number of Smears with Interpretable Results (%)** | **Number of 1+ Smears with Interpretable Results (%)** | **Number of 2+ Smears with Interpretable Results (%)** | **Number of 3+ Smears with Interpretable Results (%)** |  |
| Kinyoun | 368/391 (94.12) | 122/134 (91.04) | 125/132 (94.70) | 121/125 (96.80) |  |
| Ziehl-Neelsen | 273/428 (63.79) | 38/74 (51.35) | 44/69 (63.77) | 191/285 (67.02) |  |
| Auramine/Rhodamine | 227/383 (59.27) | 32/72 (44.44) | 77/151 (50.99) | 118/160 (73.75) |  |
| Unknown | 2/6 (33.33) | 2/2 (100.00) | 0/0 | 0/4 |  |
| **Total** | **870/1208 (72.02)** | **194 (22.30)** | **246 (28.28)** | **430 (49.43)** |  |

*Interpretable result is defined as coverage depth of 20X or greater.
