# Supplementary material for: Detection of drug resistant Mycobacterium tuberculosis by high-throughput sequencing of DNA isolated from acid fast bacilli smears
Source: PLoS One. 2020 May 8;15(5):e0232343. doi: 10.1371/journal.pone.0232343 (PMC7209238; doi:10.1371/journal.pone.0232343)
Supplement: S2 Table — (DOCX) [file pone.0232343.s006.docx]

| S2 Table. Summary of drug resistance associated mutations detected in direct AFB smears by case status and drug type. | | | | | | | | |
| --- | --- | --- | --- | --- | --- | --- | --- | --- |
|  |  |  |  |  | **Case Status** | |  |  |
| **Drug** | **Gene Segment** | **Nucleotide (Reference/Mutant)** | **Amino Acid Alteration^b^** | **New** | **Follow Up** | **Retreatment** | **Unknown** | **Total** |
| Rifampicin | *rpoB* | 1289 CTG/ CCG | L430P (L511P) | 2/703 | 0/150 | 0/40 | 0/241 | 2/1134 |
|  |  | 1295 CAA/ CCA | Q432P (Q513P) | 0/703 | 0/150 | 0/40 | 1/241 | 1/1134 |
|  |  | 1304 GAC/ GTC | D435V (D516V) | 0/703 | 1/150 | 0/40 | 1/241 | 2/1134 |
|  |  | 1333 CAC/GAC | H445D (H526D) | 38/703 | 2/150 | 1/40 | 4/241 | 45/1134 |
|  |  | 1333 CAC/TAC | H445Y (H526Y) | 2/703 | 1/150 | 7/40 | 2/241 | 12/1134 |
|  |  | 1333 CAC/AAC | H445N (H526N) | 1/703 | 0/150 | 0/40 | 0/241 | 1/1134 |
|  |  | 1349 TCG/ TTG | S450L (S531L) | 1/703 | 1/150 | 0/40 | 2/241 | 4/1134 |
|  |  | 1355 CTG/ CCG | L452P (L533P) | 0/703 | 0/150 | 0/40 | 2/241 | 2/1134 |
| **Total Rifampicin Resistant Smears (%)** |  |  |  | **44/703 (6.26)** | **5/150 (3.33)** | **8/40 (20.00)** | **12/241 (4.56)** | **69/1134 (6.08)** |
| Isoniazid | *inhA Promoter* | -15 C/T | Promoter | 3/608 | 0/137 | 0/36 | 2/234 | 5/1015 |
|  | *katG* | 944 AGC/ACC | S315T | 29/673 | 3/151 | 4/42 | 24/237 | 60/1103 |
| **Total Isoniazid Resistant Smears (%)** |  |  |  | **32/605 (5.29)** | **3/137 (2.19)** | **4/36 (11.11)** | **26/226 (11.50)** | **65/1004 (6.47)** |
| Streptomycin | *rpsL0* | 128 AAG/AGG | K43R | 13/714 | 1/155 | 3/43 | 10/257 | 27/1169 |
|  | *rpsL1* | 263 AAG/AGG | K88R | 5/647 | 0/145 | 0/40 | 3/221 | 8/1053 |
|  | *rrs10* | 513 A/C |  | 2/632 | 1/140 | 0/35 | 0/226 | 3/1033 |
|  | *rrs10* | 516 C/T |  | 2/632 | 0/140 | 0/35 | 0/226 | 2/1033 |
| **Total Streptomycin Resistant Smears (%)** |  |  |  | **22/609 (3.61)** | **2/137 (1.46)** | **3/34 (8.82)** | **13/206 (6.31)** | **40/986 (4.06)** |
| Ethambutol | *embB10* | 916 ATG/GTG | M306V | 12 | 1 | 3 | 4 | 20/1108 |
|  | *embB10* | 918 ATG/ATA | M306I | 1 | 0 | 1 | 1 | 3/1108 |
|  | *embB20* | 1190 GGC/GAC | G406D | 1 | 1 | 0 | 2 | 4/1140 |
| **Total Ethambutol Resistant Smears (%)** |  |  |  | **13/676 (2.07)^c^** | **2/145 (1.38)** | **4/40 (10.0)** | **6/239 (2.93)^c^** | **25/1100 (2.27)^c^** |
| Fluoroquinolones | *gyrA* | 281 GAC/GGC | D94G | 8/644 | 1/140 | 3/41 | 2/222 | 14/1047 |
| **Total Fluoroquinolones Resistant Smears (%)** |  |  |  | **8/599 (1.34)** | **1/134 (0.75)** | **3/35 (8.57)** | **2/198 (1.01)** | **14/966 (1.45)** |
| Moxifloxacin/ Ofloxacin | *gyrA* | 281 GAC/GGC | D94G | 8/644 | 1/140 | 3/41 | 2/222 | 14/1047 |
| **Total Moxifloxacin/ Ofloxacin Resistant Smears (%)** |  |  |  | **8/644 (1.24)** | **1/140 (0.71)** | **3/41 (7.31)** | **2/222 (0.90)** | **14/1047 (1.34)** |
| Pyrazinamide | *pncA3* | -11 A/G | Promoter | 5/615 | 1/135 | 3/37 | 1/223 | 10/1010 |
|  | *pncA3* | 11 TTG/TCG | L4S | 0/615 | 1/135 | 0/37 | 0/223 | 1/1010 |
|  | *pncA3* | 35 GAC/GCC | D12A | 0/615 | 1/135 | 0/37 | 0/223 | 1/1010 |
|  | *pncA3* | 37 TTC/CTC | F13L | 1/615 | 0/135 | 0/37 | 0/223 | 1/1010 |
|  | *pncA3* | 104 CTG/CGG | L35R | 0/615 | 0/135 | 0/37 | 2/223 | 2/1010 |
|  | *pncA3* | 137 GCA/GTA | A46V | 0/615 | 0/135 | 0/37 | 1/223 | 1/1010 |
|  | *pncA2* | 151 CAC/TAC | H51Y | 0/649 | 0/144 | 0/39 | 1/228 | 1/1060 |
|  | *pncA2* | 185 CCG/CAG; 185 CCG/CTG | P62Q; P62L Mixture | 0/649 | 0/144 | 0/39 | 1/228 | 1/1060 |
|  | *pncA2* | 188 GAC/GGC | D63G | 1/649 | 0/144 | 0/39 | 1/228 | 2/1060 |
|  | *pncA2* | 202 TGG/CGG | W68R | 1/649 | 0/144 | 0/39 | 0/228 | 1/1060 |
|  | *pncA1* | 322 GGA/AGA | G108R | 0/667 | 0/148 | 0/41 | 1/234 | 1/1090 |
| **Total Pyrazinamide Resistant Smears (%)** |  |  |  | **8/592 (1.35)** | **3/132 (2.27)** | **3/36 (8.33)** | **8/215 (3.72)** | **22/975 (2.26)** |
| ^a^ A coverage depth cut off of 20X and a frequency cut off of 80% was used for all reported single nucleotide polymorphisms. For each target gene segment, the number of smears with SNPs out of the total number of smears with 20X or greater coverage for the given target gene segment are reported by treatment status. For each category of cases and in total, the percent of smears with resistance to a given drug were calculated by dividing the number of smears with at least one mutation associated with resistance to the given drug by the total number of smears in which all relevant target gene segments (listed in Table 1) were successfully screened plus any smears in which at least one associated target gene segment met the coverage cut off and contained a drug resistance associated mutation.  ^b^ For rpoB0, the amino acid number is presented in the MTB numbering system followed by the alternative numbering system in parentheses.  ^c^ One smear with unknown treatment status and one smear from a new case contained drug resistant associated mutation in both *emb10* and *emb20* gene segments. The numerators for calculating total number of ethambutol resistant smears for new cases, cases with unknown treatment status, and overall have been adjusted to ensure we do not double count these samples. | | | | | | | | |
