# Supplementary material for: Detection of drug resistant Mycobacterium tuberculosis by high-throughput sequencing of DNA isolated from acid fast bacilli smears
Source: PLoS One. 2020 May 8;15(5):e0232343. doi: 10.1371/journal.pone.0232343 (PMC7209238; doi:10.1371/journal.pone.0232343)
Supplement: S3 Table — (DOCX) [file pone.0232343.s007.docx]

| S3 Table. Summary of non-drug resistance associated mutations detected in pncA gene of DNA isolated from direct AFB smears from Ghana, Kenya, Uganda, and Zambia. | | | | | | | |
| --- | --- | --- | --- | --- | --- | --- | --- |
| **Gene Segment** | **Nucleotide (Reference/Mutant)** | **Amino Acid Alteration** | **Ghana** | **Kenya** | **Uganda** | **Zambia** | **Total** |
| pncA3 | -46 C/G | Non-coding | 1/319 | 0/475 | 0/170 | 0/46 | 1/1010 |
| pncA3 | -32 G/A | Non-coding | 2/319 | 0/475 | 0/170 | 0/46 | 2/1010 |
| pncA3 | -12 G/A | Non-coding | 1/319 | 0/475 | 0/170 | 0/46 | 1/1010 |
| pncA3 | 45 GAG/GAT | E15D | 1/319 | 0/475 | 0/170 | 0/46 | 1/1010 |
| pncA3 | 127 CAC/TAC | H43Y | 1/319 | 0/475 | 0/170 | 0/46 | 1/1010 |
| pncA3 | 127 CAC/AAC | H43N | 1/319 | 0/475 | 0/170 | 0/46 | 1/1010 |
| pncA2 | 166 GAC/AAC | D56N | 1/327 | 0/487 | 0/199 | 0/47 | 1/1060 |
| pncA2 | 169 CAC/GAC | H57D | 3/327 | 0/487 | 1/199 | 0/47 | 4/1060 |
| pncA2 | 179 GGC/GAC | G60D | 1/327 | 0/487 | 0/199 | 0/47 | 1/1060 |
| pncA2 | 205 CCA/TCA | P69S | 27/327 | 0/487 | 0/199 | 0/47 | 27/1060 |
| pncA2 | 227 ACT/AGT | T76S | 1/327 | 0/487 | 0/199 | 0/47 | 1/1060 |
| pncA2 | 232 GGC/CGC | G78R | 1/327 | 0/487 | 0/199 | 0/47 | 1/1060 |
| pncA2 | 311 AGC/AAC | S104N | 1/327 | 0/487 | 0/199 | 0/47 | 1/1060 |
| pncA1 | 328 GAC/AAC | D110N | 1/335 | 0/496 | 0/211 | 0/48 | 1/1090 |
| pncA0 | 469 GTC/ATC | V157L | 0/333 | 0/498 | 1/209 | 0/47 | 1/1087 |
| pncA0 | 473 GAC/GGC | D158G | 1/333 | 0/498 | 0/209 | 0/47 | 1/1087 |
| pncA0 | 484 GGT/CGT | G162R | 1/333 | 0/498 | 0/209 | 0/47 | 1/1087 |
| pncA0 | 538 GTG/CTG | V180I | 1/333 | 0/498 | 0/209 | 0/47 | 1/1087 |
